# Supplementary material for: Migratory and anti-fibrotic programmes define the regenerative potential of human cardiac progenitors
Source: Nat Cell Biol. 2022 May 12;24(5):659–71. doi: 10.1038/s41556-022-00899-8 (PMC9106586; doi:10.1038/s41556-022-00899-8)
Supplement: Supplementary file 1 — Supplementary Tables 1 and 2. Gating strategies for flow cytometry analysis. [file 41556_2022_899_MOESM1_ESM.pdf]

nature portfolio

https://doi.org/10.1038/s41556-022-00899-8

Supplementary information

Migratory and anti-fibrotic programmes define the regenerative potential of human cardiac progenitors

In the format provided by the authors and unedited

Supplementary Information

1. **Supplementary Table 1**
2. **Gating strategies for flow cytometry analysis**

**1. Supplementary Table 1**<br>**List antibodies, fluorescent probes, recombinant proteins, and assays**			
**Reagent**	**Source**	**Identifier**	**Concentration (Application)**
**Antibodies**			
Anti-a-actinin, rabbit polyclonal	Abcam	ab137346	1:300 (IHC)
Anti-ACKR3, rabbit polyclonal	LSBio	LS-A1893	10 µg/ml (migration assay)
Anti-cardiac Troponin I, recombinant	Abcam	ab52862	1:500 (IHC)
Anti-Cardiac Troponin T, mouse monoclonal	Thermo Fisher Scientific	MA5-12960, Cl. 13-11	1:500 (IF)
Anti-cardiac Troponin T, rabbit polyclonal	Sigma-Aldrich	HPA015774	1:300 (IHC)
Anti-Cardiac Troponin T, rabbit polyclonal	Abcam	ab45932	1:400 (IF, flow cytometry)
Anti-CD31 (PECAM-1), sheep polyclonal	R&D systems	AF806	1:100 (IF)
Anti-CD31, rabbit polyclonal	Novus	NB100-2284	1:50 (IHC)
Anti-CD68, mouse monoclonal	eBioscience	14-0688-82, Cl. KP1	1:100 (IF)
Anti-Cleaved Caspase 3, rabbit monoclonal	Thermo Fisher Scientific	MA5-32015, Cl. SR01-02	1:100 (IF)
Anti-Collagen I, mouse monoclonal	Thermo Fisher Scientific	MA1-26771, Cl. COL-1	1:100 (IF)
Anti-CX43, rabbit polyclonal	Sigma-Aldrich	C6219	1:100 (IF)
Anti-CXCR4, mouse monoclonal	R&D systems	MAB172-SP, Cl. 44716	12 µg/ml (migration assay)
Anti-DDR2, rabbit polyclonal	Thermo Fisher Scientific	PA5-27752	1:100 (IF)
Anti-GFP, chicken polyclonal	Abcam	ab13970	1:500 (IF)
Anti-Human Nuclei, mouse monoclonal	Sigma-Aldrich	MAB1281, Cl. 235-1	1:100 (IF)
Anti-Human Nucleoli, mouse monoclonal	Abcam	ab190710, Cl.NM95	1:100 (IHC)
Anti-Integrin beta 1, mouse monoclonal	Abcam	ab24693, Cl. P5D2	10 µg/ml (migration assay)
Anti-ISL1, mouse monoclonal	DSHB	Cl. 39.4D5	1:100 (IF, flow cytometry)
Anti-Ki67, mouse monoclonal	Agilent Dako	M7240, Cl. MIB1	1:100 (IHC)
Anti-MLC2a AF647, mouse monoclonal	Synaptic Systems	311011 AT1, Cl. 56F5	1:100 IF
Anti-MLC2v, mouse monoclonal	Synaptic Systems	310111, Cl. 330G5	1:100 IF
Anti-MLC2v, rabbit polyclonal	Proteintech	10906-1-AP	1:300 (IHC)
Anti-N-cadherin, recombinant	Abcam	Ab76011	1:100 (IHC)
Anti-OCT4, rabbit polyclonal	Cell Signaling	2750	1:50 (IHC)
Anti-Periostin, rabbit polyclonal	Abcam	14041	1:100 (flow cytometry)
Anti-ROBO1, goat polyclonal	LSBio	LS-B3011	1:100 (IF)
Anti-ROBO1, goat polyclonal	R&D systems	AF1749	1:50 (flow cytometry)
Anti-ROBO1, rabbit polyclonal	Thermo Fisher Scientific	PA5-99084	5 µg/ml (signaling blockage)
Anti-SDC-4, rabbit polyclonal	Abcam	ab74139	1:500 (migration assay)
Anti-SDF-1 (CXCL12), rabbit polyclonal	Cell Signaling Technology	3740	1:100 IF
Anti-SLIT2 AF647, rat polyclonal	R&D systems	FAB5444R	1:100 (IF)

Anti-TRA-1-60, mouse monoclonal	Abcam	ab16288, Cl. TRA-1-60	1:7 (flow cytometry)
Alexa Fluor 488, goat anti mouse	Abcam	ab150113	1:250 (IF)
Alexa Fluor 488, goat anti rabbit	Abcam	ab150077	1:250 (IF)
Alexa Fluora 647, goat anti mouse	Abcam	ab150115	1:250 (IF)
Alexa Fluora 647, goat anti rabbit	Abcam	ab150079	1:250 (IF)
Alexa Fluor 594, goat anti mouse	Abcam	ab150116	1:250 (IF)
Alexa Fluor 594, goat anti rabbit	Abcam	ab150080	1:250 (IF)
Alexa Fluor 488, donkey anti chicken	Jackson Immuno Research	703-545-155	1:100 (IF)
Alexa Fluor647, donkey anti sheep	Abcam	ab150179	1:100 (IF)
Alexa Fluor 647, donkey anti goat	Abcam	ab150131	1:250 (IF)
Alexa Fluor 594, donkey anti goat	Abcam	ab150132	1:250 (IF)
Alexa Fluor 647, donkey anti rat	Abcam	ab150156	1:100 (IF)
Hoechst 33258 Staining Dye Solution	Abcam	ab228550	1:100 (IF)
**Fluorescent probes**			
Fluo-4 AM cell permeant	Thermo Fisher Scientific	F14201	3 μM (Calcium imaging)
Phalloidin (F-actin) AF647	Thermo Fisher Scientific	A22287	1:200 (IF)
WGA AF594	Thermo Fisher Scientific	W11262	1:500 (IF)
**Recombinant proteins**			
rhSDF-1 (rhCXCL12)	Peprotech	300-28A	low dose= 20 ng/ml<br>high dose= 80 ng/ml<br>(migration assay)
rhSLIT2	R&D Systems	8616-SL	2 μg/ml (signaling blockage)
**Assays**			
Click-iT EdU594 Flow Cytometry Assay Kit	Thermo Fisher Scientific	C10646	10 μM (flow cytometry)
LIVE/DEAD viability/cytotoxicity kit	Thermo Fisher Scientific	L3224	2 μM Calcein AM<br>4 μM Ethidium homodimer-1<br>(cell viability)
**Treatments**			
In solution CXCR4 Antagonist I, AMD3100	Sigma-Aldrich	239825	low dose= 50 ng/ml<br>high dose = 100 ng/ml<br>(signaling blockage)

2. Gating strategies for flow cytometry analysis

IgG Control staining

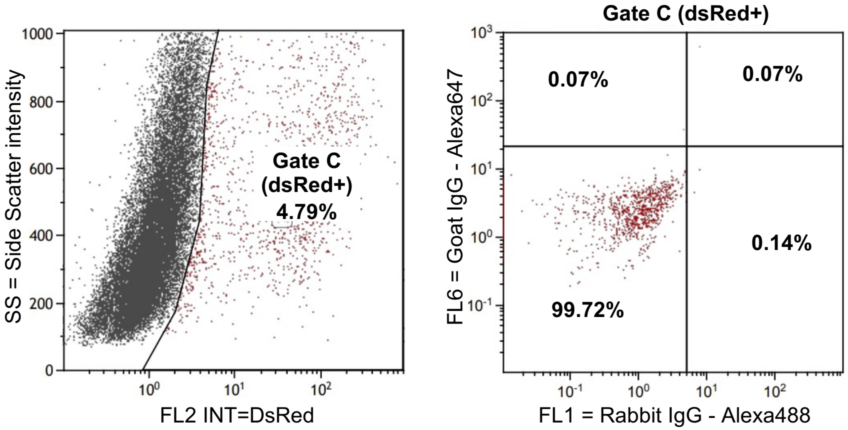

ROBO1 and POSTN staining

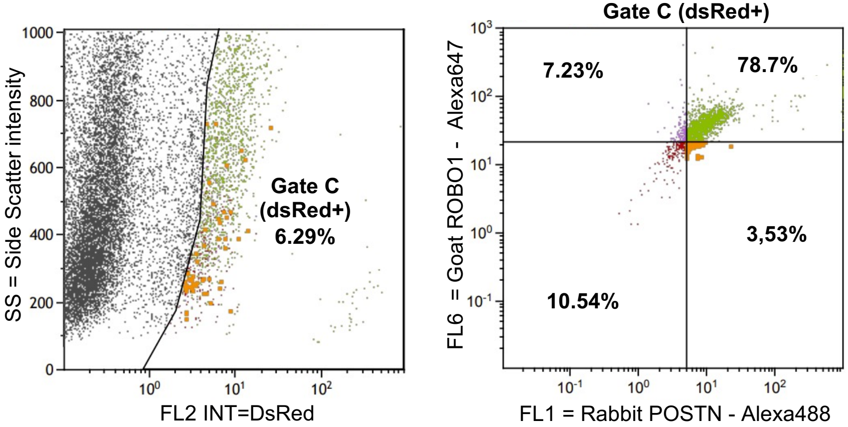

Gating strategy for flow cytometry on Figure 5f. Goat and Rabbit IgG, and relative secondary antibodies were used as negative control to set the gates on CFs^{dsRed} stained for ROBO1 and POSTN.

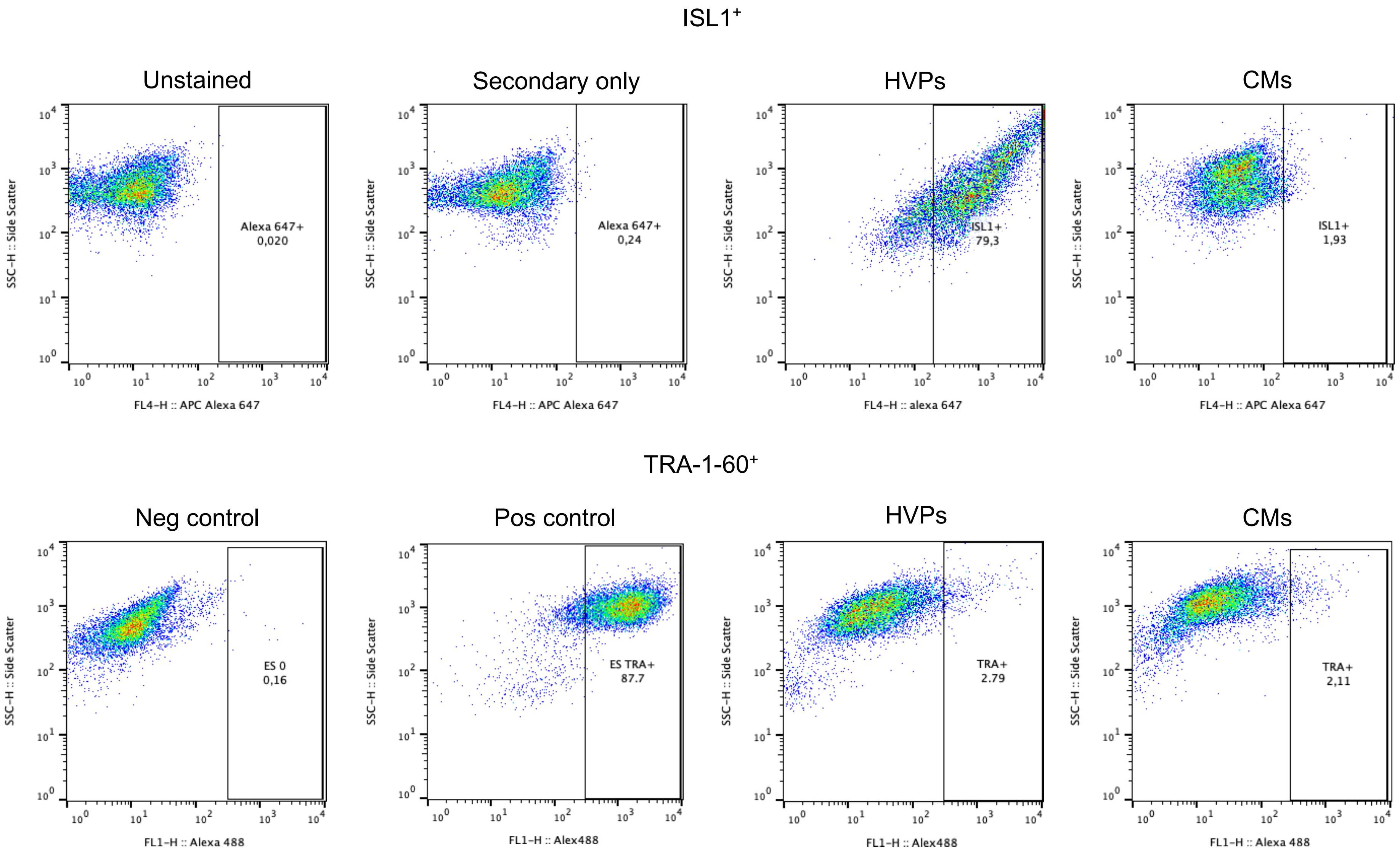

Gating strategy for flow cytometry on Extended Data Figure 3d. Unstained HVPs, secondary antibodies were used as negative control to gate for HVPs and CMs stained for ISL1 (FL4). Unstained ESCs served as negative control, and ESCs stained for TRA-1-60 were used as positive control to gate for HVPs and CMs stained for TRA-1-60 (FL1).

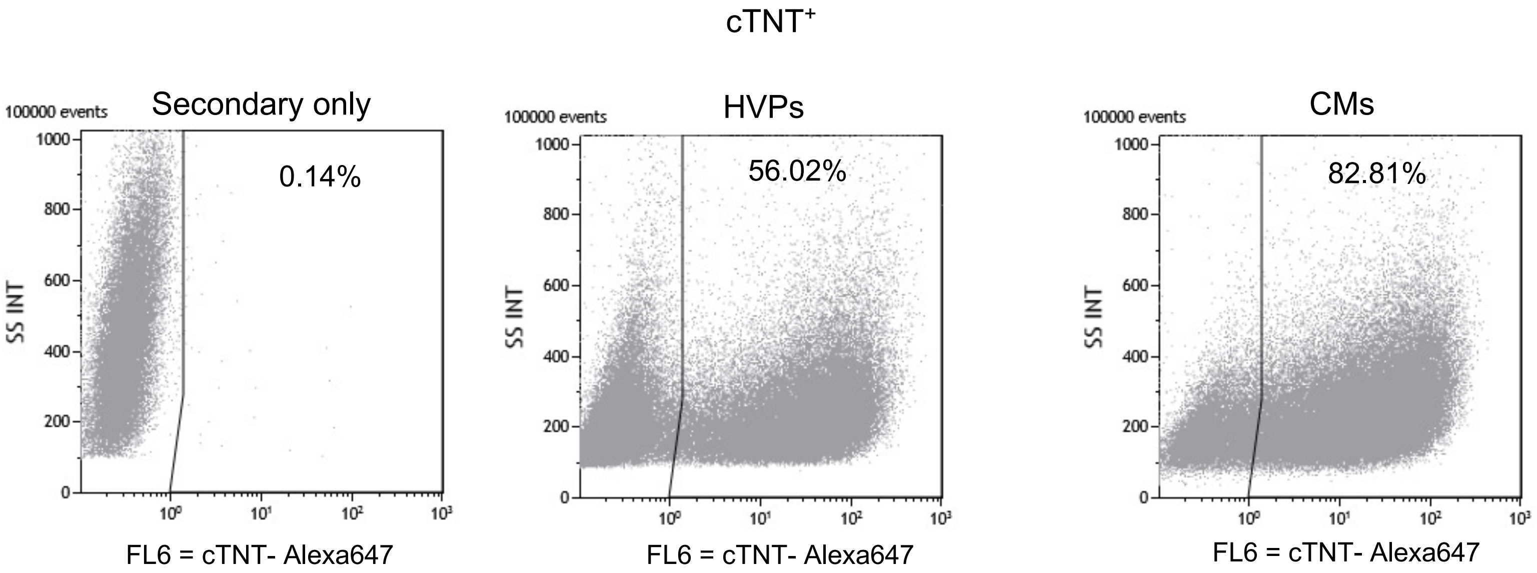

Gating strategy for flow cytometry on Extended Data Figure 3d. Secondary antibodies were used as negative control to gate for HVPs and CMs stained for cTnT (FL6).

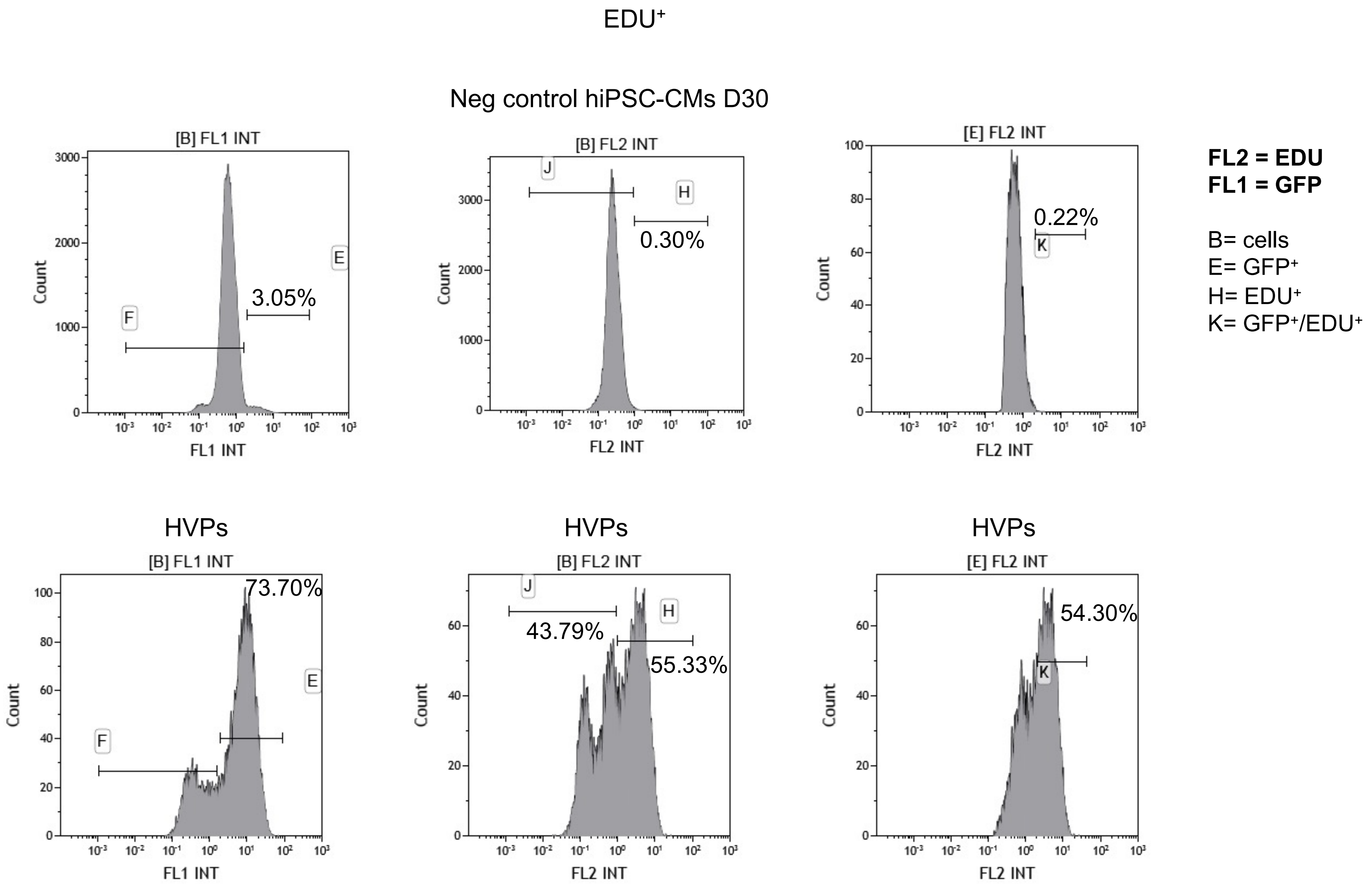

Gating strategy for flow cytometry on Extended Data Figure 1e. hiPSC-CM on D30 without fluorscent reporter were used as negative control to gate HVPs for GFP (FL1) and EDU (FL2).
