# Supplementary material for: Migratory and anti-fibrotic programmes define the regenerative potential of human cardiac progenitors
Source: Nat Cell Biol. 2022 May 12;24(5):659–71. doi: 10.1038/s41556-022-00899-8 (PMC9106586; doi:10.1038/s41556-022-00899-8)
Supplement: Supplementary file 2 — Reporting Summary [file 41556_2022_899_MOESM2_ESM.pdf]

Corresponding author(s): Christian Kupatt
Regina Fritsche-Danielson
Alessandra Moretti
Ken Chien
Karl-Ludwig Laugwitz

Last updated by author(s): Feb 15, 2022

Reporting Summary

Nature Research wishes to improve the reproducibility of the work that we publish. This form provides structure for consistency and transparency in reporting. For further information on Nature Research policies, see our Editorial Policies and the Editorial Policy Checklist.

Statistics

For all statistical analyses, confirm that the following items are present in the figure legend, table legend, main text, or Methods section.

n/a	Confirmed	
☐	☒	The exact sample size (*n*) for each experimental group/condition, given as a discrete number and unit of measurement
☐	☒	A statement on whether measurements were taken from distinct samples or whether the same sample was measured repeatedly
☐	☒	The statistical test(s) used AND whether they are one- or two-sided<br>*Only common tests should be described solely by name; describe more complex techniques in the Methods section.*
☒	☐	A description of all covariates tested
☐	☒	A description of any assumptions or corrections, such as tests of normality and adjustment for multiple comparisons
☐	☒	A full description of the statistical parameters including central tendency (e.g. means) or other basic estimates (e.g. regression coefficient) AND variation (e.g. standard deviation) or associated estimates of uncertainty (e.g. confidence intervals)
☐	☒	For null hypothesis testing, the test statistic (e.g. *F*, *t*, *r*) with confidence intervals, effect sizes, degrees of freedom and *P* value noted<br>*Give P values as exact values whenever suitable.*
☒	☐	For Bayesian analysis, information on the choice of priors and Markov chain Monte Carlo settings
☒	☐	For hierarchical and complex designs, identification of the appropriate level for tests and full reporting of outcomes
☐	☒	Estimates of effect sizes (e.g. Cohen's *d*, Pearson's *r*), indicating how they were calculated

Our web collection on statistics for biologists contains articles on many of the points above.

Software and code

Policy information about availability of computer code

Data collection	Leica application suite, Ventana Discovery Ultra autostainer V12.5.4, 1.5 Tesla Philips Intera platform R12 software
Data analysis	Kaluza V1.2, FloJo V8, , FACSAria III, Prism V9, ImageJ V1.53a, MaxQuant V1.6.2.6a, Aperio Imagescope V12.3.3.5048, MyoDish software V1, MyoDish Data File Converter V1.0, LabChart Reader V8.1.14, Rstudio Version 1.1.453, Visiopharm Software V2020.08.03.9090, Circle Cardiovascular Imaging cvi42 (V5.12)<br>RNA-seq analysis was performed in Tophat V2.1.1, Bowtie V2.4.5, R (V3.5.1), using Seurat R pipeline (Satija, R., Farrell, J. A., Gennert, D., Schier, A. F. & Regev, A. Spatial reconstruction of single-cell gene expression data. Nat. Biotechnol. 33, 495-502, doi:10.1038/nbt.3192 (2015), Pseudotemporal ordering was done using Monocle 2 (Qiu, X. et al. Single-cell mRNA quantification and differential analysis with Census. Nat Methods 14, 309-315, doi:10.1038/nmeth.4150 (2017).<br>Statistical analysis and visualization of gene sets were performed using the clusterProfiler R package (Yu, G., Wang, L. G., Han, Y. & He, Q. Y. clusterProfiler: an R package for comparing biological themes among gene clusters. OMICS 16, 284-287, doi:10.1089/omi.2011.0118 (2012)

For manuscripts utilizing custom algorithms or software that are central to the research but not yet described in published literature, software must be made available to editors and reviewers. We strongly encourage code deposition in a community repository (e.g. GitHub). See the Nature Research guidelines for submitting code & software for further information.

Data

Policy information about availability of data

All manuscripts must include a data availability statement. This statement should provide the following information, where applicable:

- Accession codes, unique identifiers, or web links for publicly available datasets
- A list of figures that have associated raw data
- A description of any restrictions on data availability

All sequencing data that support the findings of this study can be found at Gene Expression Omnibus under the accession number GSE153282.
The mass spectrometry data have been deposited to the ProteomeXchange Consortium via the PRIDE database with the dataset identifier PXD019521.
The GTF and FASTA files used for Bioinformatics analysis (GRCh37 and Mmul_1, Ensembl Releases 75 and 105, respectively) can be downloaded from Illumina iGenomes (https://emea.support.illumina.com/sequencing/sequencing_software/igenome.html).
All other data supporting the findings of this study are available from the corresponding author on reasonable request. Source data for all figures are provided with this paper.

Field-specific reporting

Please select the one below that is the best fit for your research. If you are not sure, read the appropriate sections before making your selection.

☒ Life sciences ☐ Behavioural & social sciences ☐ Ecological, evolutionary & environmental sciences

For a reference copy of the document with all sections, see nature.com/documents/nr-reporting-summary-flat.pdf

Life sciences study design

All studies must disclose on these points even when the disclosure is negative.

Sample size	The exact sample size for each experiment is provided in the figures, legends and the text.<br>For the chronic injury model, power calculation was performed with historic in-house data and data distribution determined by QQ plot analysis. For all other experiments, no statistical methods were used to pre-determine sample size. Data distribution was assumed to be normal and individual data points are presented in all graphs.
Data exclusions	No data were excluded.
Replication	All attempts for replication were successful. Unless indicated, at least 3 independant experiments were performed.
Randomization	In the porcine model, animals were evaluated based on ejection fraction after MI, and subsequently randomized to vehicle- or HVP-treatment groups. Ex vivo experiments were randomly assigned to experimental and control groups.
Blinding	cMRI was analysed blinded by two independent cardiologists.<br>In ex vivo experiments, data collection was not performed blinded due to the experimental conditions.

Reporting for specific materials, systems and methods

We require information from authors about some types of materials, experimental systems and methods used in many studies. Here, indicate whether each material, system or method listed is relevant to your study. If you are not sure if a list item applies to your research, read the appropriate section before selecting a response.

Materials & experimental systems

n/a	Involved in the study	
☐	☒	Antibodies
☐	☒	Eukaryotic cell lines
☒	☐	Palaeontology and archaeology
☐	☒	Animals and other organisms
☒	☐	Human research participants
☒	☐	Clinical data
☒	☐	Dual use research of concern

Methods

n/a	Involved in the study	
☒	☐	ChIP-seq
☐	☒	Flow cytometry
☒	☐	MRI-based neuroimaging

Antibodies

Antibodies used	Primary antibodies<br>Anti-a-actinin, rabbit polyclonal Abcam ab137346 1:300 (IHC)<br>Anti-ACKR3, rabbit polyclonal LSBio LS-A1893 10 µg/ml (migration assay)<br>Anti-cardiac Troponin I, recombinant Abcam ab52862 1:500 (IHC)

Anti-Cardiac Troponin T, mouse monoclonal Thermo Fisher Scientific MA5-12960, Cl. 13-11 1:500 (IF)
Anti-cardiac Troponin T, rabbit polyclonal Sigma-Aldrich HPA015774 1:300 (IHC)
Anti-Cardiac Troponin T, rabbit polyclonal Abcam ab45932 1:400 (IF)
Anti-CD31 (PECAM-1), sheep polyclonal R&D systems AF806 1:100 (IF)
Anti-CD31, rabbit polyclonal Novus NB100-2284 1:50 (IHC)
Anti-CD68, mouse monoclonal eBioscience 14-0688-82, Cl. KP1 1:100 (IF)
Anti-Cleaved Caspase 3, rabbit monoclonal, Thermo Fisher Scientific, MA5-32015, Cl. SR01-02, 1:100 (IF)
Anti-Collagen I, mouse monoclonal Thermo Fisher Scientific MA1-26771, Cl.COL-1 1:100 (IF)
Anti-CX43, rabbit polyclonal Sigma-Aldrich C6219 1:100 (IF)
Anti-CXCR4, mouse monoclonal R&D systems MAB172-SP, Cl.44716 12 µg/ml (migration assay)
Anti-DDR2, rabbit polyclonal, Thermo Fisher Scientific, PA5-27752, 1:100 (IF)
Anti-GFP, chicken polyclonal Abcam ab13970 1:500 (IF)
Anti-Human Nuclei, mouse monoclonal Sigma-Aldrich MAB1281, Cl. 235-1 1:100 (IF)
Anti-Human Nucleoli, mouse monoclonal Abcam ab190710, Cl.NM95 1:100 (IHC)
Anti-Integrin beta 1, mouse monoclonal Abcam ab24693, Cl. P5D2 10 µg/ml (migration assay)
Anti-ISL1, mouse monoclonal DSHB Cl. 39.4D5 1:100 (IF)
Anti-Ki67, mouse monoclonal Agilent Dako M7240, Cl. MIB1 1:100 (IHC)
Anti-MLC2a AF647, mouse monoclonal Synaptic Systems 311011 AT1, Cl. 56F5 1:100 IF
Anti-MLC2v, mouse monoclonal Synaptic Systems 310111, Cl. 330G5 1:100 IF
Anti-MLC2v, rabbit polyclonal Proteintech 10906-1-AP 1:300 (IHC)
Anti-N-cadherin, recombinant Abcam Ab76011 1:100 (IHC)
Anti-OCT4, rabbit polyclonal Cell Signaling 2750 1:50 (IHC)
Anti-Periostin, rabbit polyclonal Abcam 14041 1:100 (flow cytometry)
Anti-ROBO1, goat polyclonal LSBio LS-B3011 1:100 (IF)
Anti-ROBO1, goat polyclonal R&D systems AF1749 1:50 (flow cytometry)
Anti-ROBO1, rabbit polyclonal Thermo Fisher Scientific PA5-99084 5 µg/ml (signaling blockage)
Anti-SDC-4, rabbit polyclonal Abcam ab74139 1:500 (migration assay)
Anti-SDF-1 (CXCL12), rabbit polyclonal Cell Signaling Technology 3740 1:100 IF
Anti-SLIT2 AF647, rat polyclonal R&D systems FAB5444R 1:100 (IF)
Anti-TRA-1-60, mouse monoclonal, Thermo Fisher scientific, 14-8863-82, Cl. TRA-1-60 1:7 (flow cytometry)

Secondary antibodies:
Alexa Fluor 488, goat anti mouse Abcam ab150113 1:250 (IF)
Alexa Fluor 488, goat anti rabbit Abcam ab150077 1:250 (IF)
Alexa Fluora 647, goat anti mouse Abcam ab150115 1:250 (IF)
Alexa Fluora 647, goat anti rabbit Abcam ab150079 1:250 (IF)
Alexa Fluor 594, goat anti mouse Abcam ab150116 1:250 (IF)
Alexa Fluor 594, goat anti rabbit Abcam ab150080 1:250 (IF)
Alexa Fluor 488, donkey anti chicken Jackson Immuno Research 703-545-155 1:100 (IF)
Alexa Fluor647, donkey anti sheep Abcam ab150179 1:100 (IF)
Alexa Fluor 647, donkey anti goat Abcam ab150131 1:250 (IF)
Alexa Fluor 594, donkey anti goat Abcam ab150132 1:250 (IF)
Alexa Fluor 647, donkey anti rat Abcam ab150156 1:100 (IF)
Hoechst 33258 Staining Dye Solution Abcam ab228550 1:100 (IF)

Validation

Anti-a-actinin, rabbit polyclonal, validated for WB, ICC/IF, IHC-P in Mouse, Rat and Human
Anti-ACKR3, rabbit polyclonal, validated for IHC, IHC-P in Human, Mouse, Rat, Bat, Bovine, Dog, Hamster, Horse, Pig, Rabbit
Anti-cardiac Troponin I, recombinant Abcam, validated for Flow Cyt (Intra), WB, IP, IHC-P in Human
Anti-Cardiac Troponin T, mouse monoclonal Thermo Fisher Scientific, validated for IF/ICC, IHC (P), and IM applications in Avian, Canine, Chicken, Fish, Guinea Pig, Human, mouse, Porcine, Rabbit, and Rat
Anti-cardiac Troponin T, rabbit polyclonal Sigma-Aldrich , validated for IF in Human
Anti-Cardiac Troponin T, rabbit polyclonal Abcam, validated for IHC-P, Sandwich ELISA, WB in Human
Anti-CD31 (PECAM-1), sheep polyclonal R&D systems, validated for ICC/IF, KO, Simple Western, WB in Human
Anti-CD31, rabbit polyclonal Novus, validated for WB, ICC/IF, IHC, IHC-Fr, IHC-P in Human, Mouse, Rat, Porcine, Xenopus
Anti-CD68, mouse monoclonal eBioscience, validated for Flow, IHC(F), IHC(P), IP, WB, ICC/IF in Human, Mouse
Anti-Cleaved Caspase 3, rabbit monoclonal, Thermo Fisher Scientific, validated for WB, IHC, IF in Human
Anti-Collagen I, mouse monoclonal Thermo Fisher Scientific MA1-26771, validated for DB, ELISA, IHC, IP, WB, ICC/IF in Bovine, Deer, Human, Mouse, Pig, Rabbit, Rat
Anti-CX43, rabbit polyclonal Sigma-Aldrich, validated for ICC/IF, IHC in Hamster, Bovine, Human, Mouse, Rat
Anti-CXCR4, mouse monoclonal R&D systems MAB172-SP, validated for Flow, IHC, CyTOF, Neutralization in Human
Anti-DDR2, rabbit polyclonal, Thermo Fisher Scientific, PA5-27752, validated for WB, IHC(P), ICC/IF in Human, Mouse
Anti-GFP, chicken polyclonal Abcam ab13970, validated for WB, ICC/IF, species independent
Anti-Human Nuclei, mouse monoclonal Sigma-Aldrich MAB1281, validated for ICC, IHC, IP in Mouse
Anti-Human Nucleoli, mouse monoclonal Abcam ab190710, validated for WB, IHC-P, Flow Cyt (Intra), ICC in Human
Anti-Integrin beta 1, mouse monoclonal Abcam ab24693, validated for ICC/IF, Flow Cyt in Human. This antibody inhibits the function of beta 1 integrins and can be used to block cell adhesion (Dittell et al., 1993 and Yokosaki et al., 1994)
Anti-ISL1, mouse monoclonal DSHB validated for IHC, ICC, IF in larva, Chicken, Ferret, Fish, Frog, Human, Mouse, Rat, Zebrafish
Anti-Ki67, mouse monoclonal Agilent Dako M7240, validated for WB, IHC in Human
Anti-MLC2a AF647, mouse monoclonal Synaptic Systems 311011 AT1, validated for ICC, IHC in Human, Rat, Mouse
Anti-MLC2v, mouse monoclonal Synaptic Systems 310111, validated for WB, IP, ICC, IHC in Human, rat, mouse
Anti-MLC2v, rabbit polyclonal Proteintech 10906-1-AP, validated for WB, IP, IHC, IF in Mouse, Rat, Human
Anti-N-cadherin, recombinant Abcam Ab76011, validated for IHC, WB in Mouse, Rat, Human
Anti-OCT4, rabbit polyclonal Cell Signaling 2750 1:50, validated for WB, IHC, IF, Flow, IP in Human
Anti-Periostin, rabbit polyclonal Abcam 14041, validated for ELISA, ICC/IF, IHC-Fr, IHC-P in Mouse, Rat, Chicken, Human
Anti-ROBO1, goat polyclonal LSBio LS-B3011 1:100, validated for IHC, IHC-P, IF, WB, Peptide-ELISA in Human, Monkey, Mouse, Rat, Bat, Bovine, Dog, Hamster, Horse, Pig, Chicken, Xenopus

	Anti-ROBO1, goat polyclonal R&D systems AF1749, validated for WB, IHC in Rat<br>Anti-ROBO1, rabbit polyclonal Thermo Fisher Scientific PA5-99084 validated for WB, ICC/IF, ELISA in Human<br>Anti-SDC-4, rabbit polyclonal Abcam ab74139, validated for ELISA, WB, IHC, ICC/IF in Human<br>Anti-SDF-1 (CXCL12), rabbit polyclonal Cell Signaling Technology 3740, validated for WB in Human, Mouse, Rat<br>Anti-SLIT2 AF647, rat polyclonal R&D systems FAB5444R validated for FACS in Human, Mouse<br>Anti-TRA-1-60, mouse monoclonal, Thermo Fisher scientific, 14-8863-82, validated for WB, IHCm ICC/IFm Flow, IP, Misc in Human, Non-human primate<br><br>Secondary antibodies:<br>All secondary antibodies are validated for ICC/IF according to the manufacturers;<br>https://www.abcam.com/products?keywords=secondary%20antibodies

Eukaryotic cell lines

Policy information about cell lines

Cell line source(s)	WA09 (H9; human ES cell line) were purchased from WiCell Research Institute, USA.<br>ES03 NKX2.5GFP and H9 NKX2.5GFP cell lines were generously given to us from Dr. David Elliott (MCRI, Australia).<br>HEK293T were purchased from ATCC, USA.
Authentication	All human ES cell lines were validated in the original studies (Elliott et al., Nat Met 2011, Foo et al., Mol Ther 2018) by various means such as RNAseq, staining for pluripotency markers and determining differentiation capacity, as well as by the commercial vendors.<br>HEK293T was only used for the generation of dsRed expressing lentivirus. No additional authentication was performed.
Mycoplasma contamination	All cell lines used have been tested negatively for mycoplasma.
Commonly misidentified lines<br>(See ICLAC register)	no commonly misidentified cell line is used.

Animals and other organisms

Policy information about studies involving animals; ARRIVE guidelines recommended for reporting animal research

Laboratory animals	PIGS: For animal experiments sus scrofa, german landrace pigs, and Yucatan minipigs were used (wild-type or transgenic LEA29Y pigs); Female and male pigs were used. Mean age was 5 months, body weight ranged between 40-63kg for sus scrofa, german landrace pigs. Mean age was over 6 months, body weight ranged between 28-40 kg for Yucatan minipigs.<br>NHPs: NHP slices were obtained after termination of control animals that were part of various studies from primate centers in Göttingen, at the Karolinska Institute or the LMU Munich.
Wild animals	study did not involve wild animals
Field-collected samples	study did not involve samples collected from the field
Ethics oversight	PIGS: All animal experiments with german landrace pigs were performed with permission of the local regulatory authority, Regierung von Oberbayern (ROB), Munich, (approval number: AZ 02-18-134). Applications were reviewed by the ethics committee (Sachgebiet 54) according to §15 TSchG German Animal Welfare Law.<br>All Yucatan minipig experiments were approved by IACUC (Institutional Animal Care and Use Committees) under the study number 1974-061 in Charles River Mattawan (MI, USA).<br><br>NHPs: German primate centre, Göttingen (file reference: 33.19-42502-04-16/2264), Karolinska Institutet, Sweden (file reference: N 277/14) and Walter Brendel Institute, LMU, Germany (file reference: ROB-55.2--2532.Vet_02-14-184).

Note that full information on the approval of the study protocol must also be provided in the manuscript.

Flow Cytometry

Plots

Confirm that:

☒ The axis labels state the marker and fluorochrome used (e.g. CD4-FITC).

☒ The axis scales are clearly visible. Include numbers along axes only for bottom left plot of group (a 'group' is an analysis of identical markers).

☒ All plots are contour plots with outliers or pseudocolor plots.

☒ A numerical value for number of cells or percentage (with statistics) is provided.

Methodology

Sample preparation	For baseline expression of ISl-1, cTNT and Tra-1-60, human ES cells were differentiated into ventricular progenitor cells, in vitro. On D6 and D25, cells were dissociated with accutase before staining for desired epitopes according to manufacturer's instructions.

Instrument	FACS Canto III, Gallios flow cytometer (Beckman Coulter, USA)
Software	FloJo software version 8 (BD Biosciences), Kaluza software version 1.2 (Beckman Coulter, USA)
Cell population abundance	Purity of sample was determined with FACS Canto III or Galios flow cytometer, and it is dependent on markers to markers.
Gating strategy	Gating was done in light of negative control (secondaries only or unstained control cells).

☒ Tick this box to confirm that a figure exemplifying the gating strategy is provided in the Supplementary Information.
